# Estimating the Directional Spread of Epidemics in Their Early Stages Using a Simple Regression Approach: A Study on African Swine Fever in Northern Italy

**DOI:** 10.3390/pathogens12060812

**Published:** 2023-06-07

**Authors:** Vincenzo Gervasi, Marco Sordilli, Federica Loi, Vittorio Guberti

**Affiliations:** 1 Istituto Superiore per la Protezione e la Ricerca Ambientale, Via Ca’ Fornacetta, 9, 40064 Ozzano Emilia, Italy; 2 Direzione Generale Sanità Animale e Farmaci Veterinari, Ministero della Salute, Via Giorgio Ribotta, 5, 00144 Roma, Italy; 3 Osservatorio Epidemiologico Veterinario Regionale della Sardegna, Istituto Zooprofilattico Sperimentale della Sardegna, 07100 Sassari, Italy; federica.loi@izs-sardegna.it

**Keywords:** *Asfaviridae*, carcasses, early detection, regression models, disease surveillance, *Sus scrofa*, wild boar

## Abstract

The early identification of the spreading patterns of an epidemic infectious disease is an important first step towards the adoption of effective interventions. We developed a simple regression-based method to estimate the directional speed of a disease’s spread, which can be easily applied with a limited dataset. We tested the method using simulation tools, then applied it on a real case study of an African Swine Fever (ASF) outbreak identified in late 2021 in northwestern Italy. Simulations showed that, when carcass detection rates were <0.1, the model produced negatively biased estimates of the ASF-affected area, with the average bias being about −10%. When detection rates were >0.1, the model produced asymptotically unbiased and progressively more predictable estimates. The model produced rather different estimates of ASF’s spreading speed in different directions of northern Italy, with the average speed ranging from 33 to 90 m/day. The resulting ASF-infected areas of the outbreak were estimated to be 2216 km^2^, about 80% bigger than the ones identified only thorough field-collected carcasses. Additionally, we estimated that the actual initial date of the ASF outbreak was 145 days earlier than the day of first notification. We recommend the use of this or similar inferential tools as a quick, initial way to assess an epidemic’s patterns in its early stages and inform quick and timely management actions.

## 1. Introduction

The early identification of an epidemic infectious disease is an important first step towards implementing effective interventions and to reduce the resulting mortality and morbidity in target populations [1]. Early detection strictly depends on the surveillance methods put in place, particularly when a disease affects wild populations rather than livestock animals [2]. In such cases, animal host movements and social structures are also crucial in disease transmission and spatial dynamics [3,4]. Often, epidemics are well under way before the authorities are notified and epidemic control measures are put in place. On the other hand, implementing adequate surveillance activities is neither simple nor inexpensive. Thus, the robust and cost-effective estimation of a disease’s spreading patterns in term of distance, speed, and directionality is necessary for successful early detection and to have a chance of stopping the disease’s spread.

African swine fever (ASF) is considered the most serious animal disease that the world has had for a long time, not only for its high mortality and animal health consequences [5] but for the measureless economic losses reported in all countries where the virus is present [6]. This disease, caused by a large, double-stranded DNA virus (ASFV), currently affects both domestic and wild pig populations [7]. Some differences, though, have been reported in ASF spreading patterns between Europe and Asia. In the Asian continent, the disease mainly affects domestic pigs, and its introduction in farms is mostly attributable to human behaviour [8]; in Europe, the current spread of ASF genotype II mainly affects the wild boar population, which subsequently represents the main risk factor for disease introduction in domestic pig farms, given the low biosecurity [8]. Even if most European countries seem to manage ASF outbreaks in pig farms reasonably well, the gradual ASFV spread in wild boar populations at the local scale is very difficult to control and often persists over long periods [1].

After its introduction in Georgia in 2007, ASFV genotype II reached the territory of the European Union in 2014 [9]. Despite intensive awareness campaigns against ASF and continuous control efforts based on studies of the infection’s dynamics, ASFV has progressively spread in a western direction, affecting several EU countries [8,9]. Among them, in December 2021, ASF genotype II also reached northern Italy [10] and, in particular, Liguria and Piedmont, where the virus continues to cause multiple cases in wild boars; in May 2022, a second introduction of ASF—likely independent—was recorded in the municipality of Rome, where wild boars again acted as an epidemiological reservoir for the virus, which was also detected in an outbreak in domestic pigs (June 2022) [11].

As was previously demonstrated, the long-term persistence of ASF at low wild boar densities is strictly related to direct and carcass-mediated infection [12,13]. Thus, the early detection of ASF during the initial spread of the virus in a susceptible population, as well as the early detection and removal of infected wild boar carcasses, can be crucial in influencing the evolution of the epidemic. Moreover, when ASF is identified for the first time in a European country, several control measures must be put in place following the European Commission’s (EC) legislation. Specifically, target actions on both domestic pig and wild boar populations are managed based on the identification of an infected zone and a surveillance zone, which define the spatial context in which movement and hunting restrictions, active surveillance in domestic pig farms, the search and removal of active wild boar carcass, the installation of fences, and intensive wild boar depopulation are enforced [13,14]. The borders of the infected area are generally defined by the spatial distribution of all ASF cases (mostly infected wild boar carcasses) reported since the initial disease outbreak [15]. One limitation of this approach is that there is often a delay between the epidemiological evolution of the disease and the ability for a surveillance system to detect new cases in previously unaffected areas [9]. Therefore, the estimated infected area is suspected of being an underestimation of the actual area at any given point in time. Moreover, the ASF spatial dynamics in the landscape are not static. The ASF front changes and expands over time, and the speed of such an expansion process is usually not the same in all directions because of local variations in wild boar density or the presence of geographical barriers to animal movement [16]. The most widespread methods for estimating the speed of a disease outbreak are usually based on either the evaluation of the frequency distribution of all case-to-case distances in space and time or on a linear regression between the linear distance and the timespan of each case from the initial outbreak location. While simple to implement, these methods provide just a single estimate of the outbreak speed, failing to reveal the differential dynamics in the different directions in space. On the other hand, more mechanistic approaches, such as the ones using spatially explicit individual-based models, can predict the spatial evolution of a disease in different directions and reveal the underlying causes of disease spreading. In a recent review [17], Hayes et al. reported 34 scientific studies using mechanistic models to explore ASF spatial dynamics. These approaches, though, are characterised by a high level of structural complexity and require many input parameters, which are, in most cases, unavailable, especially in the early stages of an outbreak. More recent works [18] have used the principles of diffusion models, which are traditionally applied to the exploration of expansion processes in gases and other physical systems by means of Brownian motion theories. These approaches have the advantage, with respect to mechanistic approaches, of requiring a rather limited number of parameters. On the other hand, the applications presented so far did not explore the directional component of the diffusion process.

In this work, we present a generic, regression-based method that allows for the estimation of the directional speed of ASF expansion in its early epidemiological phases, using data from passive surveillance, particularly the location and date of the field identification of all ASF-infected carcasses in a newly affected area. We assessed the performance of our method through a simulation design. Then, the real data on notified ASFV cases in the first Italian cluster of Piedmont and Liguria (PL hereafter) were used in a case study to validate the model, providing a practical example of its potential applicability to management purposes. The scope of this study was not to reach the same level of insight and understating of ASF spatial dynamics that can be produced by more complex, mechanistic approaches, but to present a simple predictive tool that could inform decision makers and support them in zoning processes. Typically, a simple model of the directional spreading of ASF could help define where restrictions and surveillance efforts should be focused during the early phases of an ASF outbreak, thus increasing the chances of early detection and early eradication.

## 2. Materials and Methods

The rationale behind our analytical design was to choose a simple statistical approach that could be applied with a limited dataset and that had enough flexibility to improve the understanding of ASF spreading patterns in its early stages, thus providing guidance in surveillance and control efforts. To this aim, we developed a simple protocol for collecting the necessary temporal and spatial variables from the data; then, we selected a subset of ASF cases suitable for the estimation of ASF speed; finally, we applied a generalised additive model (GAM, [19]) to produce the directional estimates of ASF spreading. This method is especially suited to making use of data derived from passive surveillance programs, i.e., the spatial and temporal distribution of ASF-positive carcasses.

### 2.1. Model Building and Evaluation through Simulations

To produce the necessary simulated dataset, we used a spatially explicit, individual-based model of wild boar demography and ASF epidemiology, which allowed us to generate a realistic spatial and temporal distribution of the ASF-positive carcasses from an initial outbreak location. The model is described in detail in [12]. We ran the model on a population of 24,300 wild boar, distributed on a simulated 8100 km^2^ area, corresponding to a density of three wild boar/km^2^. A single infected individual was placed in the centre of the simulated area on day zero. To generate a differential speed of the ASF front in the different directions, we defined transmission rates as a function of the angle between the location of a potential transmission event and the initial outbreak location, according to the following function:

$$logit(\beta_{x})=\beta_{0}+\beta_{1}\times \cos\left(x\right)+\beta_{2\;}\times \sin\left(x\right)$$

in which *β_x_* was the directional transmission rate corresponding to angle *x*, *β*_0_ was the average transmission rate, as defined in [12], while *β*_1_ and *β*_2_ were the two direction-specific correction factors, related to the sine and cosine of angle *x*, expressed in radians. At each simulation, the values of *β*_1_ and *β*_2_ were randomly and independently selected in the range 0–1, to allow for all the possible combinations of strength and directionality in the ASF spread.

We ran the individual-based model for 250 days, during which we also simulated the probability for each ASF-infected carcass to be detected by the passive surveillance system. We simulated four scenarios of increasing detection rates, ranging from 10% to 40%. At the end of the simulated period, we extracted the location and day of detection for all the ASF-infected carcasses retrieved by the surveillance system, thus mimicking the typical dataset available in the field. For each data point, we calculated the distance and the angle, with respect to the initial outbreak location. Then, we identified a subset of all the retrieved carcasses, to be used for the actual estimation of the ASF directional speed. Not all transmissions, in fact, correspond to an advancement of the disease front. In each direction and for each day, only those transmissions occurring farther from the initial outbreak than any other, contribute to increase the disease front. All the transmissions occurring behind the front should be, therefore, disregarded. To this aim, we divided the 360° range into 20 bins, each covering a 18° angle. Such choice was subjective and corresponded to a compromise between the resolution of the directional estimates and the requirement to have enough data points in each bin. Any other choice could be applied to other applications of the method, depending on the number of data points available. After assigning each data point to its respective bin, we kept only those carcasses whose distance from the initial outbreak location was larger than those of any other carcass detected inside the same bin at an earlier date. This assured that all the data points used for subsequent analyses represented a step forward in the advancement of the disease front.

Once the dataset was defined, we used the distance from each carcass to the initial outbreak location as a response variable in the GAM model after checking its normal distribution; to explain the variation in the response variable, we applied three smoothing factors to the cosine and sine of angle *x* and to the number of days between the initial outbreak and the day of carcass detection. The use of additive modelling allowed us to capture non-linear relationships between the variables and to identify local minima and maxima in the effects, thus providing additional flexibility for the model’s application in the real-world, where local spatial factors (such as geographical barriers or corridors) can strongly limit or favour the possibility of the epidemic front moving towards a certain direction. We performed the statistical modelling in R 4.2.1 [20]. For each simulated scenario, we ran 100 iterations and summarised the resulting parameter estimates.

After running all models, we used the estimated regression coefficients of all iterations to generate the predicted ASF spreading speed in each of the 20 directions and to predict the distance from the initial outbreak location to the ASF front at 250 days in each direction. The resulting minimum convex polygon (MCP_model_) corresponded to the model-based estimate of the ASF-affected area at 250 days from the outbreak start. To assess model performance, for each scenario and iteration we also constructed two additional MCPs: (i) one derived from all the ASF-infected carcasses (detected and non-detected) in the simulated study area during the 250-day study period (MCP_real_), whose size corresponded to the real ASF-infected area; (ii) one derived only from the detected carcasses, whose size corresponded to the estimated size of the ASF-infected area resulting from the raw field data (MCP_field_), without the use of any modelling approach. The relative bias of the modelling approach in estimating the ASF-affected area was calculated as (MCP_model_ − MCP_real_)/MCP_real_, whereas the bias associated with the field data was calculated as (MCP_field_ − MCP_real_)/MCP_real_. A comparison of these two quantities among the different scenarios allowed us to evaluate the advantages of using a modelling approach with respect to the simple identification of an affected area based on field data.

### 2.2. Application to the ASF Outbreaks in Northern Italy

**Figure 1:**
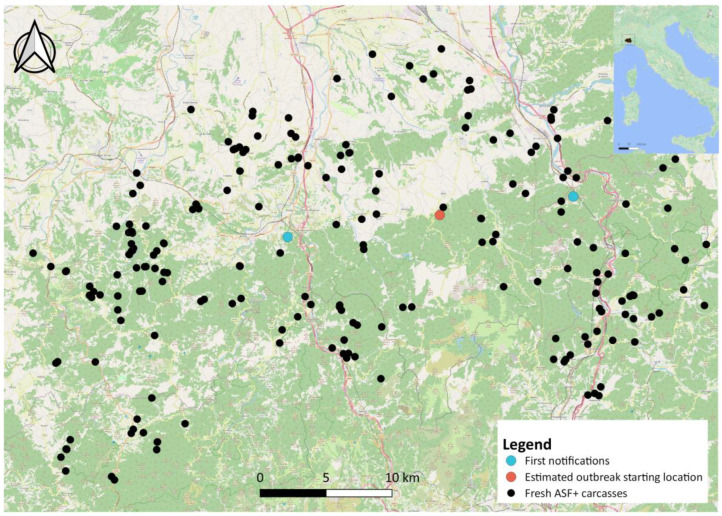
Spatial distribution of the ASF-infected carcasses detected in northwestern Italy between 29 December 2021 and 28 February 2023, used to estimate the directional spread of ASF. The two locations of the first ASF notifications are highlighted in light blue, whereas the average location between these two initial carcasses is shown in red.

After evaluating the method on simulated data, we applied it to the real case of the first ASF outbreak on wild boar in northwestern Italy (Figure 1). Field data were collected using the Veterinary Information Systems of the Italian Ministry of Health (VETINFO), and using the national animal disease database for passive surveillance (SINVSA). The following recorded information was related to all the wild boar tested for ASFV: location (region, province, municipality, latitude, and longitude); the date of sampling; the date of notification if the presence of ASFV was confirmed by PCR+; the age and sex of the tested animal; the type of sample (i.e., spleen, blood, tonsil, kidney, or lymph node); and whether the sample arose from breeding, carcasses, or wild boar killed by road traffic. Carcass condition was established during sample collection based on the stage of conservation, and it was recorded as fresh, in decomposition, or in advanced decomposition according to the FAO manual on ASF in wild boar [5].

The ASF outbreak in northwestern Italy emerged through two distinct infected carcasses, identified on the same day (29 December 2021) about 25 km from each other (Figure 1), as previously described in [11]. From a spatial point of view, we considered the average location between these two carcasses as the potential centroid of the ASF spread and analysed the spatial and temporal dynamics of the disease using all ASF-positive carcasses detected between 29 December 2021 and 28 February 2023 (426 days). As one of the main issues when using passive surveillance data is the potential bias related to carcasses’ different statuses of conservation (i.e., fresh, in decomposition, mummified), we included in the analysis only the ASF PCR+ carcasses noted as fresh during sample collection, which amounted to 232 carcasses [11].

**Figure 2:**
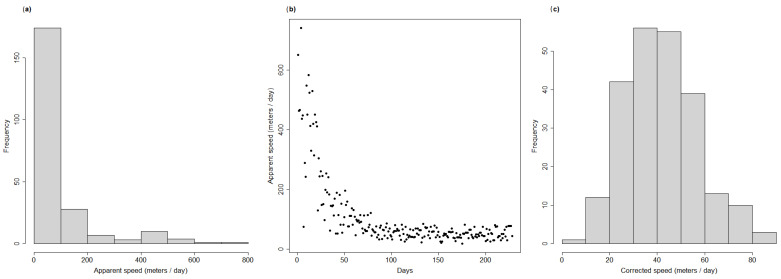
Frequency distribution of the apparent speed associated with all the fresh, ASF-infected carcasses detected in northwestern Italy between 29 December 2021 and 28 February 2023 (**a**); the relationship between apparent speed and the number of days since the first ASF notification is also illustrated in (**b**). The corrected speed of the ASF notifications is also illustrated in (**c**), after accounting for the potential bias induced by the use of a midpoint as a possible initial location of the ASF outbreak.

As was performed for the simulated dataset, for each wild boar carcass we extracted the distance, time difference and angle with respect to the cluster initial location, and removed those carcasses detected behind the disease front, thus obtaining the final dataset for analysis (n = 90). One potential issue when calculating the number of days between the detection of each carcass and the initial outbreak day was that the actual outbreak day was likely to have been earlier than the day of first notification (29 December 2021). This is suggested by the presence of very large apparent speed values for a few carcasses in the dataset (Figure 2a), and by the fact that all the extremely high apparent speed values were concentrated in the first few days after the first two ASF notifications (Figure 2b).

To correct for such possible bias, and to try and estimate the most likely day of actual disease outbreak, we built a generalised linear model (GLM) with a Poisson distribution between the apparent speed associated with each carcass and the number of days since the first ASF notification. We removed one outlier from the dataset, corresponding to an apparent speed of about 2 km in a single day (Figure 2b). After estimating model parameters, we projected the model back in time and estimated on which day the model predicted an apparent speed equal to that of the first carcass. This provided us with an estimate of the most likely day on which the ASF epidemic started. Then, we used this estimate to recalculate the number of days between each carcass’ notification and the estimated start of the outbreak. The histogram of the recalculated speed of propagation for each case is reported in Figure 2c, and it shows that, after accounting for the distortion effect, the distribution became unimodal and normal in its shape. Finally, similar to what was done for the simulated datasets, we ran a GAM model using two smooths for the sine and cosine variables and applied a third smooth on the number of days since the start of the outbreak.

As a preliminary validation of the selected models, we visually examined the linear relationship between the fitted and response values and evaluated the ratio between the effective degrees of freedom (EDF) and the basis dimension *k*. When EDF and *k* had similar values, the model exhibited a low ability to describe emerging trends in the data. Additionally, we performed a *k*-fold cross validation [21] as a more formal way to assess the predictive power of the two models. We selected 80% of the dataset from each cluster to estimate regression parameters based on the best selected model; then, we generated predicted values for the remaining 20% of the data and assessed the Pearson’s correlation index between observed and predicted values. We repeated the validation randomly over 100 iterations and calculated the accuracy score as the median correlation index over all iterations of each of the two models.

## 3. Results

### 3.1. Model Evaluation through Simulations

**Figure 3:**
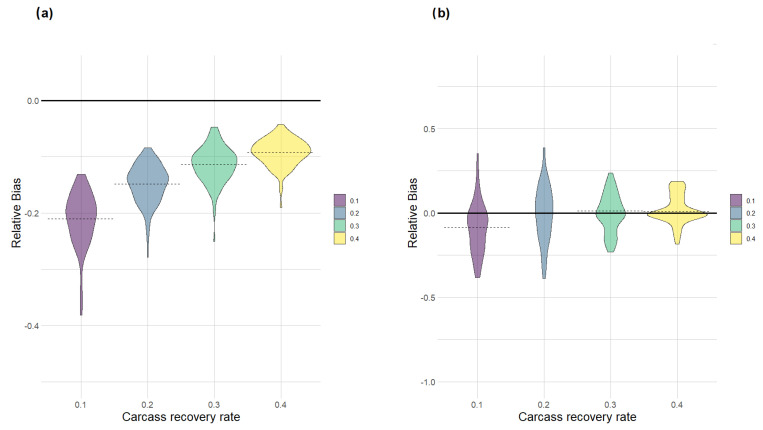
Accuracy in estimating the size of the ASF-infected area using the simple bounding of all detected locations (**a**) and using an inferential method based on a regression-based approach using the date and location of the same infected carcasses (**b**). The relative bias is shown for a range of increasing carcass detection rates. Dashed lines indicate the average bias associated with each scenario.

As expected, the use of field-collected carcasses in a simulated environment produced an underestimation of the ASF-infected area at 250 days, especially when the proportion of infected carcasses was low. Field-based estimates of the ASF- infected area were in average 20% smaller than the actual size when carcass recovery rate was set to 0.1 (Figure 3a); the average bias was reduced to about 10% when increasing carcass detection rates to 0.4, but higher detection rates never totally removed the systematic negative bias in the estimation of the affected area (Figure 3a). As illustrated in Figure 3b, the modelling approach resulted in an improvement of the estimates in all simulated scenarios. When carcass detection rates were low (*p* = 0.1), the GAM model still produced slightly biased estimates of the ASF-affected aera, with the average bias being around −10%. For higher values of the carcass detection rate (*p* > 0.1), the estimator became asymptotically unbiased and progressively more predictable (Figure 3b); when 40% of all infected carcasses were detected, about 80% of the estimates had a bias lower than 10%.

### 3.2. Application to the ASF Outbreaks in Northwestern Italy

During the study period, a total of 12,001 wild boar were sampled from the whole Italian territory, of which 4172 were from the Piedmont-Liguria infected areas (Figure 1). A total of 419 ASF cases were reported in this study area during the 426-day period. Of these, 299 were from carcasses (Table 1).

As reported in Table 1, 1914 (46%) of the samples collected were females. Through a dentition evaluation, as described by Matsche in 1967 [22], most of the sampled wild boar in the PL area were determined to be adults (n = 1896, 45%), and 66% (n = 2745) of the samples tested for ASF were from passive surveillance, collected during field search activities (n = 1773) or from road traffic accidents (n = 972), while 34% (n = 1427) of the samples tested were from active surveillance. In total, 3290 (79%) carcasses had a fresh conservation status, whereas 17% (n = 729) were in decomposition, and 2% (76) were mummified. Of the 419 ASF PCR+ wild boar, 50% (n = 209) were adults.

Overall, 232 samples, referred to as PCR+ fresh carcasses, were considered for the purpose of this work. Only 90 PCR+ fresh carcasses contributed to the advancement of the disease front and were included in the final analysis. As a test of sensitivity for this choice, we also ran a model including all 232 reported carcasses, but the model performed poorly, as the estimated ASF-affected area was smaller than the observed one, something which reveals a serious degree of underestimation in model parameters. This confirmed that the choice of only using carcasses identified beyond the ASF front at any time was correct.

**Figure 4:**
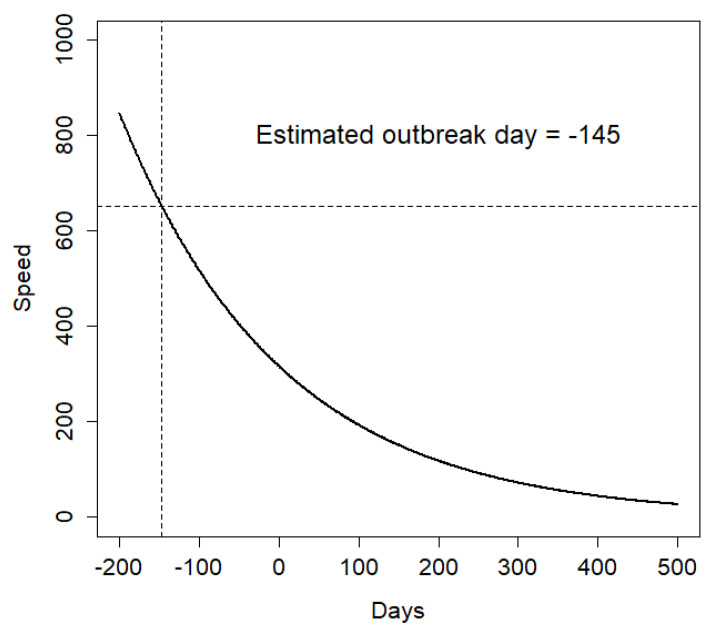
Relationship between the number of days since the first ASF notification and the apparent speed associated with each carcass, as estimated using a Poisson GLM model. The estimated outbreak day, corresponding to an apparent speed of 652 m/day, was 6 August 2021.

The Poisson GLM regression revealed a significant negative relationship between the number of days since the first ASF notification and apparent speed (β = −0.0049, SE = 0.0004, *p* < 0.001). The apparent speed associated with the first carcass was 652 m/day. The model predicted that such apparent speed was likely to be the result of an initial ASF outbreak occurring 145 days before the first ASF notification, i.e., on 6 August 2021 (Figure 4). We used this estimated date as the new ASF outbreak day for all subsequent analyses.

The GAM model, applied to the resulting dataset, revealed that all the smoothers had a significant effect on the dependent variable, suggesting that the speed of ASF propagation changed in space and time. For all smoothers, the number of effective degrees of freedom was lower than the selected *k* parameter (*k* = 9), confirming that the choice of *k* was appropriate. The model had an R^2^ = 0.77 and was able to explain 80.9% of the observed variance. The *k*-fold cross validation also performed relatively well, with the average correlation between observed and predicted values being 0.87.

**Figure 5:**
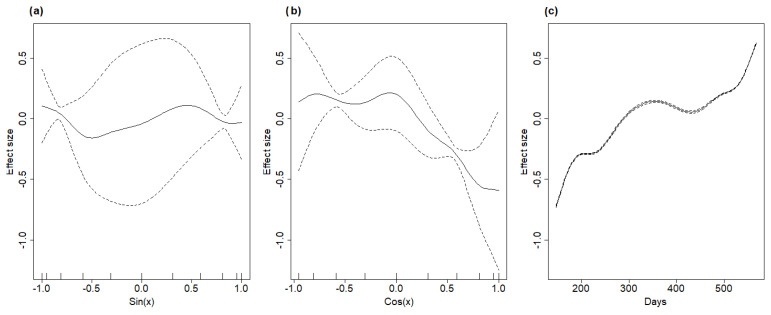
Effects of the sine (**a**) and cosine (**b**) of the angle between an ASF+ carcass and the initial outbreak location on the speed of propagation of the disease front. The effects of the number of days is also shown (**c**).

The model produced rather different estimates for ASF spreading speed in the different directions (Table 2, Figure 5a,b). At its maximum, ASF spreading speed was estimated to be 90 m/day (SE = 71.1; 95% CIs = 71–106); at its minimum, towards west, this was estimated to be 33 m/day (SE = 4.1; 95% CIs = 25–41).

**Figure 6:**
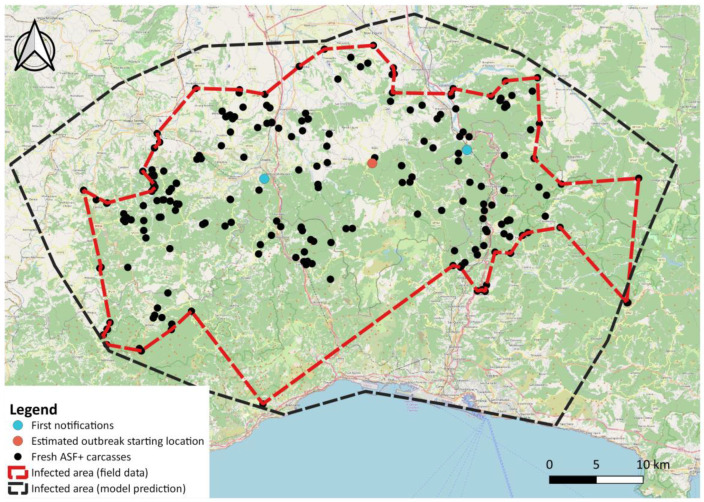
Spatial distribution of the ASF-infected carcasses detected in northwestern Italy between 29 December 2021 and 28 February 2023, used to estimate the ASF directional spread. The two locations of first ASF notification are highlighted in light blue, whereas the average location between these two initial carcasses is shown in red. The dashed red polygon indicates the ASF-affected area, based on the distribution of all detected ASF-positive carcasses. The black, dashed polygon indicates the ASF-infected area, as estimated through a GAM model.

The resulting ASF-infected area was estimated to be 2216 km^2^, about 80% bigger than the one identified only through field data (Figure 6).

## 4. Discussion

The results of our simulation exercise showed the high performance of the estimation method, especially when evaluated in relation to the limited amount of information required to estimate the directional speed of propagation. The model had unbiased behaviour when carcass detection rates were low, but not extremely low (10–30%). This is the case in most of the surveillance programs put in place so far for ASF in Europe [23]. On the other hand, the estimation method becomes less and less justified when detection rates increase to high values (>40%), as the estimated infected area overlaps more and more with the area identified by simply connecting all the retrieved carcasses in a certain period.

It should be noted, though, that in our simulations, we did not test the spatial variation in detection rates or its consequences on the performance of the estimator. In this sense, the model is not expected to be robust to this type of variation. Hence, if the efforts toward carcass search and removal are very low in certain areas or directions, the model will underestimate the speed of propagation for that portion of the infected area. This stresses the need for keeping efforts in passive surveillance as uniform as possible both in space and in time. The active search of infected wild boar carcasses should not be driven by the distribution of those carcasses already found, but it should be a priori determined inside an area of interest and kept as constant as possible, regardless of the actual number of ASF-positive carcasses retrieved [24,25]. Alternatively, the effort expended in carcass detection should be recorded during passive surveillance activities to be used as an additional covariate in the model and to prevent biased estimates of ASF speed due to local differences in sampling efforts.

For the specific case of the ASF outbreaks in northwestern Italy, our model performed reasonably well given the small sample size, as both the R^2^ values and the results of the *k*-fold cross validation were overall satisfactory. Moreover, the analysis of the apparent ASF speed suggested that the actual start of the outbreak might have occurred up to 5 months earlier than the date of first notification, as supported by evidence that the two first carcasses, identified on the same day, were 20 km apart. Moreover, it is promising that the estimator was able to detect large differences in the ASF speed depending on the geographical direction, despite the limited number of data points available. We should stress that airborne infection was not involved in disease transmission, because this is not the case for the ASFV, and that human factors are expected to have played a minor role in defining the disease front, as no hunting or other forest activity was allowed after the disease outbreak in all of the affected area.

The estimated infected area was about 80% larger than the one defined by simply considering the bounding polygon of all the identified carcasses. This result is in line with the patterns revealed in the simulation exercise, and it confirms that surveillance is often behind the epidemiological development of the disease. This is a crucial element, because the early identification of where the disease front lies at any point in time is a fundamental requirement for acting timely through restrictions and other management actions. We believe that the application of an inferential approach, although simple, can help reduce this lag between epidemiology and management, and increase the chances for managers to act rapidly and timely in the first phases of an outbreak. Modelling studies [26] and the field experiences of the only two successful ASF eradications in Europe (Belgium and Czech Republic, [27]) both indicate that the first weeks after an ASF outbreak are the period during which eradication chances are the highest, if effective actions are implemented when the infected area is still relatively small. Once ASF has spread into an area large enough to prevent an effective fencing, its eradication becomes very difficult [27]. In this context, any analytical tool that can help identify the infected area and the expected spreading patterns in a quick but accurate way should be used to inform the decision-making process.

It is also interesting to note that the model produced rather different estimates of the ASF speed of propagation as a function of time (Figure 5c), showing an initial phase of rapid diffusion in winter 2021/2022, followed by a slower dynamic during summer and by a new rapid increase during winter 2022/2023. This highlights that such a predictive tool, although simple, can provide some insight both in the spatial and in the temporal component of the ASF spreading process. On one hand, information regarding the speed of disease propagation in different directions can support managers when they are expected to produce zoning maps that define exclusions, limitations, and surveillance protocols. The current homogeneous buffering system does not consider that the disease can move faster in certain directions than others, thus leaving the management system behind the outbreak evolution. On the other hand, estimating how ASF speed varies in time can support the distribution of surveillance efforts during the year, as in some periods of the year, ASF is expected to generate a significantly higher density of infected carcasses than in others. Moreover, the results presented in Figure 5c suggest that, in northern Italy, the period of the year with the highest virus circulation might be winter, which differs from what has been observed in other countries, such as the Baltic states and Poland. One possible explanation for such a difference might be the difference in the average seasonal temperatures between the two geographical contexts. Summer temperatures in southern European and Mediterranean areas could reduce virus persistence on wild boar carcasses and in the environment, thus producing a reduction in the overall infection probability, whereas winter temperatures might be more compatible with a longer persistence and higher circulation of the ASF virus. This calls for an evaluation of how the epidemiological dynamics of ASF could differ in the Mediterranean area, with respect to what has been observed and estimated for the Eastern and Northern European countries, based on the expected differences in both wild boar density and climate.

The main limitation of a relatively simple analytical approach like the one presented here, is that it provides no insight into the underlying reasons for the spreading patterns but just describes it in space and time under the assumption of a flat, homogeneous landscape. One example of such a limitation is represented in the southwestern corner of the estimated ASF-affected area in Figure 6. This area, where no ASF cases were found during the study period, corresponds to the city of Genoa, where, although the number of wild boars present in the city itself is very high, the virus apparently never arrived. The absence of the virus could be explained by the fact that the city is separated from the forest system by several stretches of motorway and highways that slow down or prevent the natural spatial spread of the virus. More than 300 wild boars were sampled in the area, most of them in active surveillance, all of which tested ASF negative. While the additive regression approach could be easily extended with the inclusion of new variables, such as the existence of topographic barriers in certain directions, local differences in host densities or in search effort, more mechanistic and data-hungry approaches, such as step selection functions [28], should be used to couple the estimation of the speed of propagation with a deeper understanding of the epidemiological dynamics driving the spatial patterns. Additionally, while our simulation exercise provides evidence of how the model is expected to perform under a range of sampling conditions, new, real-world applications of existing ASF surveillance datasets is crucial for testing how such a simple approach, which provided meaningful information for the northwestern Italian context, could be successfully applied to other geographical and ecological situations.

Finally, the economic aspects associated with carcass search and removal should also be considered in effective surveillance. Decision makers should consider the social context and the resources needed/available in terms of the associated costs. To be successful, passive surveillance requires the use of an intensive effort for a prolonged period, a scenario that might be not affordable for all countries currently affected by the ASFV. This partly represents a limitation in the application of our estimation method, as the data needed to build and analyse it and to estimate the directional spread of the disease can only be produced with substantial economic investment in wild boar monitoring and disease surveillance. Additionally, as ASF has no limitations against moving and spreading across countries, a lack of international collaboration and data sharing could limit the applicability of this and other inferential methods in border areas between two or more countries. Data standardisation and sharing should be seen as a crucial objective to improve our ability to understand disease dynamics at a large scale and to respond promptly to new emerging outbreaks.

## Figures and Tables

**Table 1:** Summary statistics of all the wild boar samples collected and analysed in the Piedmont-Liguria region during the ASF outbreak period. Statistics Refers to culled or hunted animals or those killed by road traffic accidents. Following the EFSA definition, passive surveillance only refers to animals found dead and not those culled by road traffic accidents [15].

Feature of the Samples	Piedmont-Liguria Infected Area (n = 4172)
Female	1914 (46%)
Age	
Young (0–6 months)	961 (23%)
Subadult (6–18 months)	1153 (28%)
Adult (>18 months)	1896 (45%)
NA	162 (4%)
Source of the sample	
Active surveillance	1427 (34%)
Passive surveillance	2745 (66%)
Carcasses conservation stage	
Fresh	3290 (79%)
In decomposition	729 (17%)
Advanced decomposition	76 (2%)
NA	77 (2%)
PCR+ samples	419 (10%)
Young (0–6 months)	47 (11%)
Subadult (6–18 months)	101 (24%)
Adult (>18 months)	209 (50%)
NA	62 (15%)
PCR+ fresh carcasses	232 (55%)

**Table 2:** Direction-specific estimates of the ASF speed of propagation and of the predicted distance between the initial outbreak location and the disease front at the end of the study period (28 February 2022).

Angle	ASF Front after 426 Days (km)	Directional Speed (m/Day)
0° (eastward)	33.2	79
36°	20.3	48
72	16.9	40
108°	13.9	33
144°	21.5	51
180° (westward)	37.9	90
216°	33.9	81
252°	28.6	68
288°	34.9	83
324°	32.0	76

## Data Availability

The data used for the analyses are publicly available at https://izsam.maps.arcgis.com/apps/dashboards/bc047a485e68458d83a61e48b4f8c20a, accessed on 6 June 2023.
